# Differential Roles of Three α-Crystallin Domain-Containing sHsps of *Beauveria bassiana* in Asexual Development, Multiple Stress Tolerance and Virulence

**DOI:** 10.3390/ijms23126717

**Published:** 2022-06-16

**Authors:** Gang Zhou, Sheng-Hua Ying, Ming-Guang Feng, Jie Wang

**Affiliations:** 1 College of Food Science, South China Agricultural University, Guangzhou 510642, China; zgbees@gdim.cn; 2 Guangdong Provincial Key Laboratory of Microbial Culture Collection and Application, State Key Laboratory of Applied Microbiology Southern China, Institute of Microbiology, Guangdong Academy of Sciences, Guangzhou 510070, China; 3 Institute of Microbiology, College of Life Sciences, Zhejiang University, Hangzhou 310058, China; yingsh@zju.edu.cn

**Keywords:** *Beauveria bassiana*, small heat shock proteins, asexual development, environmental adaptation, virulence

## Abstract

Small heat shock proteins (sHsps) containing conserved α-crystallin domain play important roles in many cellular processes, but little is known about the functions of sHsps in filamentous entomopathogens. Here, three sHsps of Hsp20, Hsp30a, and Hsp30b were characterized in *Beauveria bassiana*, a filamentous fungal insect pathogen that serves as the main source of wide-spectrum fungal insecticides. The results demonstrated that these three genes are interrelated at the transcriptional level under normal and heat-shocked conditions. Meanwhile, all the deletion mutants showed significant but differential changes in cell wall integrity, antioxidant activity, hyphal tolerance to carbendazim fungicide, conidial tolerance to 45 °C wet heat and virulence. However, only *Δhsp30b* showed growth defects on rich and minimal media at 25 °C and *Δhsp30a* displayed the reduction in conidiophores and conidia. Moreover, the single deletion of *hsp30a* and *hsp30b* caused the decreases in hyphal growth at 34 °C and conidial tolerance to UV-B irradiation. Our findings provide a global insight into vital roles of *hsp20*, *hsp30a*, and *hsp30b* in asexual development, environmental adaptation, and fungal virulence of *B. bassiana*.

## 1. Introduction

Heat shock proteins (Hsps) are a family of stress proteins that are synthesized by cells when exposed to stressful conditions [1]. According to their molecular sizes, Hsps can be classified into six families, namely Hsp100, Hsp90, Hsp70, Hsp60, Hsp40, and small Hsps [2]. Among them, small Hsps exist widely in all kingdoms of life and display less sequence conservation between species. Generally, small Hsps were characterized by a small molecular weight of 12–43 kDa and a conserved α-crystallin domain of about 90 residues flanked by variable N- and C-terminal extensions in sequence and length [3,4,5]. In *Saccharomyces cerevisiae*, the sHsp system consists of Hsp26 and Hsp42 [6]. The phenotypes of *Δhsp26* in *S. cerevisiae* demonstrated that this gene had no effect on the spore development and germination, cellular growth at various temperatures, thermoresistance during sporulation, and tolerance to ethanol, but is important for the cell morphology under normal and high temperatures [7]. However, *S. cerevisiae Δhsp42* cells showed abnormal morphology [6,8]. In *Candida albicans*, only one sHsp with α-crystallin domain was identified, namely Hsp21 [9]. The disruption of *hsp21* resulted in various phenotypic changes, including the defects in hyphal formation and cellular growth under conditions of nutrient limitation, the decrease in the resistance to heat shock and kinds of chemical stressors (i.e., menadione, ethanol and dithiothreitol), the delay in germ tube formation under the stress of Hsp90 inhibitor, and reduced pathogenicity, which suggests Hsp21 contributes to thermotolerance, adaptation to oxidative stress, ethanol-induced stress, and antifungal drugs, growth under conditions of nutrient limitation, hyphal formation, and fungal virulence in *C. albicans* [9]. In addition, the deletion of *hsp21* prevented phosphorylation of Cek1 at elevated temperatures and affected the homeostasis of glycerol under osmotic stress, glycogen under oxidative stress, and trehalose under thermal stress [9]. In *Ustilago maydis*, Hsp20, only one sHsp containing α-crystallin domain, positively regulated the fungal virulence and fungal tolerance to hydrogen peroxide [10]. In *Neurospora crassa*, inactivation of *hsp30*, encoding an α-crystallin-related heat shock protein, showed poor survival under the combined stresses of heat shock and the restriction of glucose and accumulated unphosphorylated glucose at high temperature by reducing the hexokinase activity [11,12]. These studies indicated that fungal sHsps played important roles in fungal growth and development, thermotolerance, adaption to chemical stresses, and virulence but functionally differentiated in different genes and fungi. However, the functions of sHsps have not been explored in filamentous entomopathogens.

*Beauveria bassiana*, a classic filamentous entomopathogenic fungus, has been commercially severed as the main source of many eco-friendly mycoinsecticides [13]. The active ingredients of fungal insecticides are composed of conidia, blastospores and/or mycelia, whose survival is often affected by natural and man-made stress factors affecting the field stability and persistence of biocontrol agents, such as high temperature, solar ultraviolet radiation, and fungicides [14]. Therefore, it is necessary to understand the molecular mechanisms involved in, and hence explore effective means to improving, the environmental adaptation of fungal cells. Previously, two Hsp40 proteins of Mas5 and Mdj1 contributed to conidiation capacity, insect pathogenicity, and conidial heat tolerance as well as played important roles in cellular responses to UV-B irradiation, high osmolarity, oxidation and/or cell wall perturbation and in transcription and translation of many phenotype-related genes in *B. bassiana* [15,16]. In addition, six Hsp70s were proved to be important for biological control potential of *B. bassiana* due to differential roles in the maintenance of cell wall integrity, antioxidant activity and cellular cation homeostasis [17]. These limited findings demonstrated the essential roles of Hsps in fungal growth, adaptation to multiple environmental stresses, and virulence. The genomic sequencing analysis showed *B. bassiana* possesses 28 Hsps consisting of 3 sHsps, 6 Hsp40s, 1 Hsp60, 15 Hsp70s, 1 Hsp90, and 2 Hsp100s [13]. However, aside from two Hsp40s and six Hsp70s, the functions of most Hsps remained unknown. This study sought to analyze the roles of small Hsps containing α-crystallin domain in sustaining the fungal growth and development, multiple stress tolerance, and virulence based on the differences of multiple phenotypes among wild type (WT) strains, single-gene disruption mutants, and complemented strains.

## 2. Results

### 2.1. Bioinformatic Features of sHsps in B. bassiana

Three sHsps, namely Hsp20 (NCBI accession codes: EJP63081), Hsp30a (EJP62362), and Hsp30b (EJP69022), containing the α-crystallin domain were found in *B. bassiana* genome [13] with the queries of *S. cerevisiae* Hsp26 and Hsp42. Hsp20, Hsp30a, and Hsp30b consist of 432, 197, and 232 amino acids with molecular masses of 47.04, 21.78, and 25.72 kDa and isoelectric points of 6.45, 6.05, and 5.49, respectively, and were predicted to locate in the cytoplasm. Comparisons of the amino acid sequences and a phylogenetic analysis revealed that *B. bassiana* Hsp20, Hsp30a, and Hsp30b share 25.66–47.41% sequence identity with one another and 22.88–93.91% sequence identity with 23 other Hsp20s or Hsp30s in fungi and Hsp20 was subdivided into the ScHsp26 clade whereas Hsp30a and Hsp30b were subdivided into the ScHsp42 clade (Figure S1A).

### 2.2. Interrelationships of hsp20, hsp30a, and hsp30b and Their Roles to Heat Tolerance

**Figure 1:**
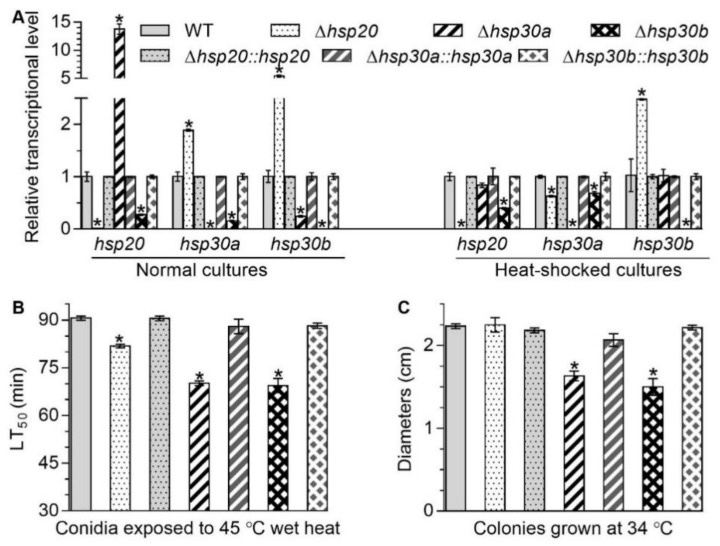
Changes in transcriptional profiles of *hsp20*, *hsp30a*, and *hsp30b* and responses to high temperature caused by the deletion of *hsp20*, *hsp30a*, and *hsp30b* in *B. bassiana*. (**A**) Relative transcript levels of *hsp20*, *hsp30a*, and *hsp30b* in the 3-day-old SDAY cultures of each mutant exposed or not exposed to 40 °C for 1 h. (**B**) LT_50_ for conidial tolerance to 45 °C wet heat. (**C**) Diameters of fungal colonies after 8 days of cultivation in SDAY at 34 °C. Asterisked bars in each bar group differ significantly from those unmarked (Tukey’s HSD, *p* < 0.05). Error bars: SD from three replicates.

The deletion and complemented mutants of *hsp20*, *hsp30a* and *hsp30b* were identified and confirmed by PCR (Figure S1B). Additionally, the transcript level of *hsp20*, *hsp30a*, or *hsp30b* was undetectable in the corresponding deletion mutant and well restored to WT levels in the complemented mutants according to quantitative real-time PCR (qRT-PCR) analyses (Figure 1A), providing another evidence for the success of each deletion and complementation.

Deletion of one sHsp gene altered the transcript level of two others with respect to the WT standard when all of mutants were cultured on the plates of Sabouraud dextrose agar plus yeast extract (SDAY) under normal and heat-shocked conditions. In *Δhsp20*, the transcript level of *hsp30a* increased by 88.78% under normal condition but decreased by 37.42% under heat-shocked condition and the transcript level of *hsp30b* increased by 444.09% and 147.95% under normal and heat-shocked conditions, respectively, compared with that in the WT (Figure 1A). In *Δhsp30a*, the transcript level of *hsp20* and *hsp30b* were upregulated by 1275.73% and 75.77%, respectively, under normal condition but showed no significant change under heat-shocked conditions, compared with that in the WT (Figure 1A). In *Δhsp30b*, the transcript level of *hsp20* and *hsp30a* were reduced respectively by 72.62% and 84.42% under normal condition and by 60.39% and 31.94% under heat-shocked condition, respectively, compared with that in the WT (Figure 1A). These data suggested that these three sHsp genes in *B. bassiana* were conditionally interrelated at the transcriptional level.

Compared with the WT, median lethal times (LT_50_s; min) for conidial tolerance to a wet-heat stress at 45 °C in *Δhsp20*, *Δhsp30a*, and *Δhsp30b* were shortened by 9.68%, 22.57%, and 23.38%, respectively (Figure 1B), and mean diameters of fungal colonies in *Δhsp30a* and *Δhsp30b* decreased by 26.86% and 32.84%, respectively, during 8-day incubation at 34 °C (Figure 1C). However, the absence of *hsp20* did not affect significantly the fungal colony diameters at 34 °C (Figure 1C). These data indicated that these three sHsps were differently responsive to thermal stress in *B. bassiana*.

### 2.3. Differential Roles of hsp20, hsp30a, and hsp30b in Radial Growth and Conidiation

**Figure 2:**
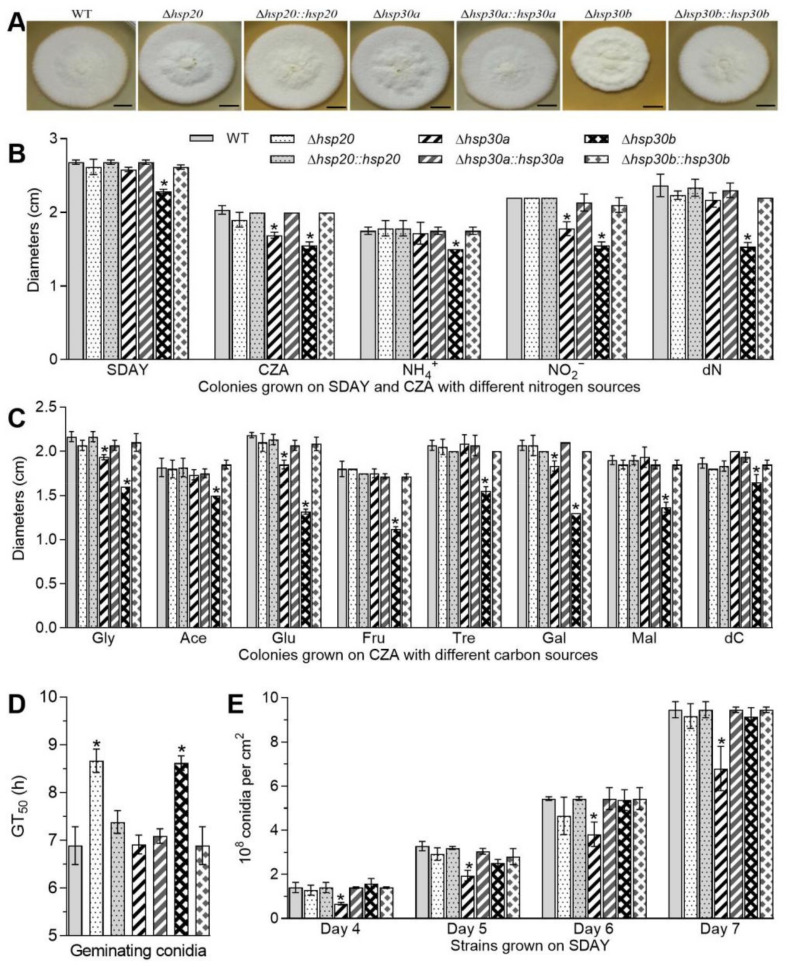
Differential roles of *hsp20*, *hsp30a*, and *hsp30b* in the asexual cycle of *B. bassaiana*. (**A**) Images of fungal colonies after 8-day growth on SDAY plates at 25 °C. (**B**,**C**) Diameters of fungal colonies after 8 days of cultivation at 25 °C on SDAY, CZA and modified CZA media with different carbon/nitrogen sources. (**D**) GT_50_s on germination medium (GM) plates. (**E**) Conidial yields during the period of incubation. Asterisked bars in each bar group differ significantly from those unmarked (Tukey’s HSD, *p* < 0.05). Error bars: SD from three replicates.

The single deletion of *hsp20*, *hsp30a*, and *hsp30b* resulted in differential defects in vegetative growth, conidial germination, and conidiation capacity. Compared with the WT, only *Δhsp30b* showed growth defects on SDAY plates and its colony diameter was shortened by 14.91% (Figure 2A,B). Moreover, most severe growth defects on minimal Czapek-Dox agar (CZA) and 11 modified CZA media with different carbon or nitrogen sources were also observed in *Δhsp30b*, whose colony diameter decreased by 11.61–39.69% after 8-day incubation (Figure 2B,C). The colony diameter of *Δhsp30a* diminished by 16.89% and 19.15% on the nitrogen sources of NO^3−^ and NO^2−^, respectively, and by 10.77% and 15.27% on the carbon sources of glycerol and glucose, respectively, but were not significantly affected on other tested media. However, the deletion of *hsp20* did not cause any growth defects on SDAY, minimal CZA and 11 modified CZA media (Figure 2B,C). Additionally, GT_50_s for 50% of conidial germination of *Δhsp20* and *Δhsp30b* conidia at 25 °C was significantly longer (delay for 1.78 and 1.73 h, respectively) than that of the WT (Figure 2D), but there was no significant difference between conidial GT_50_s of *Δhsp30a* and the WT.

Conidial yields quantified from 4-, 5-, 6-, and 7-day-old SDAY cultures reached 1.40, 3.28, 5.44, and 9.46 × 10^8^ conidia cm^−2^ on average in the WT, respectively, and were reduced by 51.86%, 40.77%, 29.71%, and 28.14% in *Δhsp30a*, respectively but was not affected by the deletion of *hsp20* or *hsp30b* (Figure 2E). Accompanied by the defects in conidial yields, *Δhsp30a* showed much fewer conidiophores and conidia than the WT in microscopic examination of samples taken from colonies on day 3 (Figure S2).

The defects in radical growth, conidial germination, and conidiation capacity caused by the single deletion of *hsp20*, *hsp30a*, and *hsp30b* were well restored in the complementary mutants. These results demonstrated that among the three sHsp genes, *hsp30a* significantly contributed to fungal conidiation and *hsp30b* was important for vegetative growth in *B. bassiana*.

### 2.4. Essential Roles of hsp20, hsp30a, and hsp30b in Cell Wall Integrity

**Figure 3:**
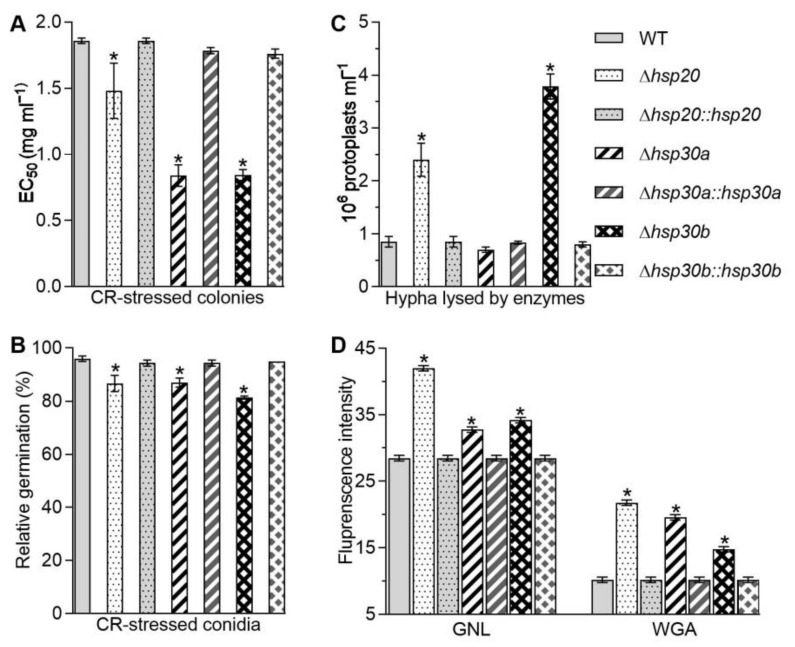
Contributions of *hsp20*, *hsp30a*, and *hsp30b* to cell wall integrity of *B. bassiana*. (**A**) EC_50_ for Congo red to suppress 50% colony growth of each strain after 6 d incubation at 25 °C on 1/4 SDAY plates. (**B**) Relative germination percentages of conidia on the GM plate alone and supplemented with Congo red (1 mg/mL) after 24 h incubation at 25 °C. (**C**) Concentrations of protoplasts released from the hyphal cells after 6 h treatment with cell wall lysing enzymes in osmotic solution of 0.8 M sucrose. (**D**) Fluorescence intensity from flow cytometry of 2 × 10^4^ conidia labeled with the fluorescent lectins GNL and WGA. Asterisked bars in each bar group differ significantly from those unmarked (Tukey’s HSD, *p* < 0.05). Error bar: SD from three replicates.

The *Δhsp20*, *Δhsp30a*, and *Δhsp30b* mutants decreased fungal tolerance to Congo red, a cell wall perturbing agent, during colony growth and conidial germination. As a consequence of a 6-day incubation with Congo red, the effective concentrations (EC_50_s) of the concentration required to inhibit colony growth by 50% in *Δhsp20*, *Δhsp30a*, and *Δhsp30b* were lowered by 20.36%, 54.83%, and 54.61%, respectively, compared with the estimate of WT (Figure 3A). The relative germination percentages of conidia on GM plates supplemented with Congo red also decreased by 9.72% in *Δhsp20*, 9.38% in *Δhsp30a*, and 15.28% in *Δhsp30b* in comparison to 96% on average in the WT (Figure 3B). Moreover, more protoplasts released from hyphal cells were observed in *Δhsp20* and *Δhsp30b* than that in the WT after 6-h treatment with cell lysing enzymes (Figure 3C). Compared with the WT, the number of released protoplasts increased by 182.35% and 345.20% in *Δhsp20* and *Δhsp30b*, respectively, but showed no significant difference in *Δhsp30a* (Figure 3C), suggesting the absence of *hsp20* and *hsp30b* but not *hsp30a* resulted in increased fragility of hyphal wall. Furthermore, the content of galanthus nivalis lectin (GNL)-labeled mannose residues increased by 47.39%, 15.09%, and 20.00% on the conidial surfaces of *Δhsp20*, *Δhsp30a*, and *Δhsp30b*, respectively, compared with the WT (Figure 3D). The content of wheat germ agglutinin (WGA)-labeled β-GlcNAc and sialic acids on the conidial surfaces of *Δhsp20*, *Δhsp30a*, and *Δhsp30b* increased by 113.84%, 92.25%, and 45.14%, respectively, compared with the WT (Figure 3D). The results from the fluorescent lectin-binding assays indicated that the single deletion of *hsp20*, *hsp30a*, and *hsp30b* altered the composition of conidial wall. Finally, images observed under transmission electron microscopy showed that outermost layer of conidial wall became less distinctly outlined in all deletion mutants than that in the WT (Figure S3).

The increases in fungal tolerance to Congo red, the number of the released protoplasts, and the content of cell wall composition in the deletion mutants were largely or well restored by the targeted gene complementation, indicating vital but differential roles for *hsp20*, *hsp30a*, and *hsp30b* in sustaining the cell wall integrity in *B. bassiana*.

### 2.5. Contributions of hsp20, hsp30a, and hsp30b to Stress Tolerance and Virulence

**Figure 4:**
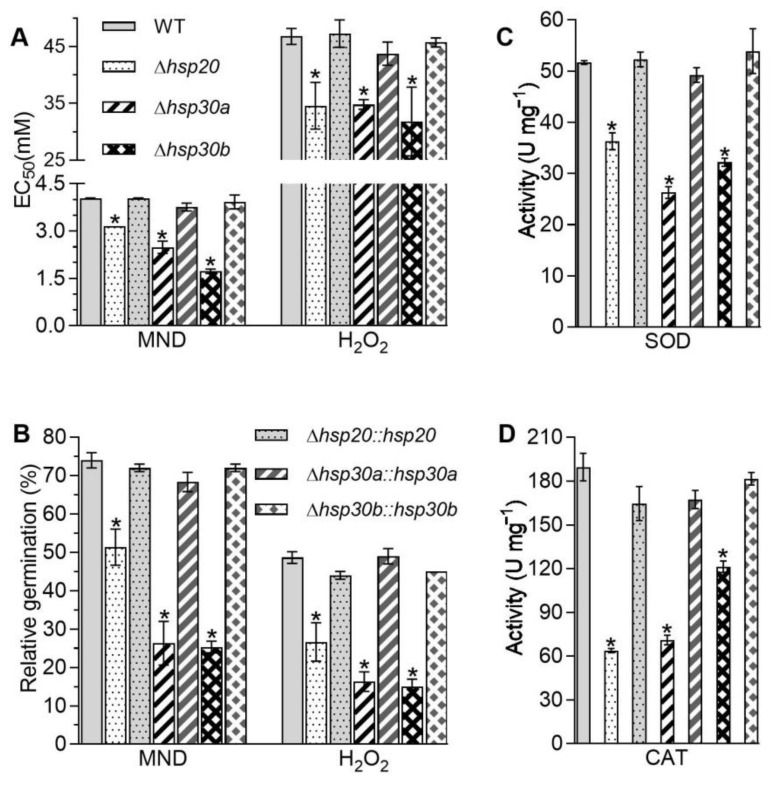
Essential roles of *hsp20*, *hsp30a*, and *hsp30b* in cell response to oxidative stress. (**A**) EC_50_s for menadione (MND) and H_2_O_2_ to suppress 50% colony growth of each strain after 6 d incubation at 25 °C on 1/4 SDAY plates. (**B**) Relative germination percentages of conidia on the GM plate alone and supplemented with MND (0.2 mM) and H_2_O_2_ (4 mM) after 24 h incubation at 25 °C. (**C**,**D**) Total SOD and CAT activities quantified in the protein extracts from 3-day SDAY cultures. Asterisked bars in each bar group differ significantly from those unmarked (Tukey’s HSD, *p* < 0.05). Error bar: SD from three replicates.

The single deletion of *hsp20*, *hsp30a*, and *hsp30b* resulted in the increases in sensitivities to the oxidants menadione and H_2_O_2_ during colony growth and/or conidial germination. The EC_50_ values for menadione and H_2_O_2_ required for 50% suppression of hyphal growth were lowered by 21.62% and 26.09% in *Δhsp20*, 38.32% and 25.62% in *Δhsp30a*, and 57.20% and 32.03% in *Δhsp30b*, respectively, compared with those in the WT (Figure 4A). The percentages of conidial germination in response to menadione and H_2_O_2_ were also lowered by 30.63% and 45.21% in *Δhsp20*, 64.41% and 66.44% in *Δhsp30a*, and 65.77% and 69.18% in *Δhsp30b*, respectively in comparison to 74% and 49% on average in the WT, respectively (Figure 4B). Moreover, compared with those in the WT, the total activities of superoxide dismutases (SODs) required for removal of menadione-generating superoxide anions and catalases (CATs) required for H_2_O_2_ decomposition were reduced by 29.78% and 66.35% in *Δhsp20*, 49.13% and 62.54% in *Δhsp30a*, and 37.74% and 36.04% in *Δhsp30b*, respectively (Figure 4C,D).

**Figure 5:**
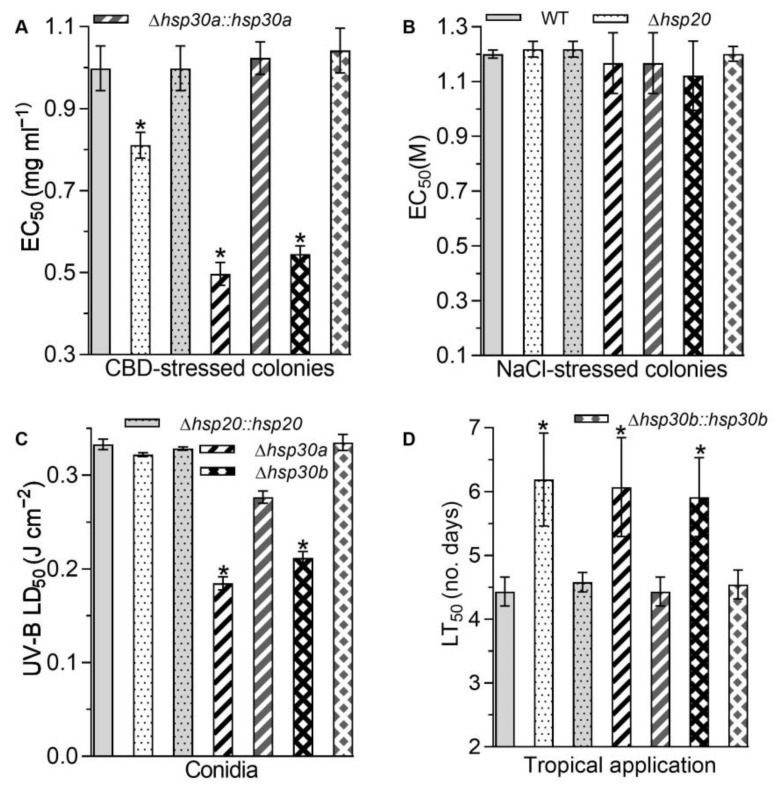
Phenotypic changes in response to carbendazim (CBD), NaCl, and UV-B irradiation and fungal virulence caused by the deletion of *hsp20*, *hsp30a*, and *hsp30b* in *B. bassiana.* (**A**) EC_50_ for CBD to suppress 50% colony growth of each strain after 6 d incubation at 25 °C on 1/4 SDAY plates. (**B**) EC_50_ for NaCl to suppress 50% colony growth of each strain after 6 d incubation at 25 °C on 1/4 SDAY plates. (**C**) LD_50_ estimates for conidial resistance to UV-B irradiation. (**D**) LT_50_ for the virulence of each strain against *G. mellonella* larvae infected by topical application. Asterisked bars in each bar group differ significantly from those unmarked (Tukey’s HSD, *p* < 0.05). Error bar: SD from three replicates.

In addition to the altered sensitivities to two oxidants, the single deletion of *hsp20*, *hsp30a*, and *hsp30b* led to the increase in the sensitivities to carbendazim, a fungicide. Based on the EC_50_ reduction relative to the WT estimate, hyphal tolerance to carbendazim decreased by 18.78%, 50.25%, and 45.33% in *Δhsp20*, *Δhsp30a*, and *Δhsp30b*, respectively (Figure 5A). However, the deletion of *hsp20*, *hsp30a*, and *hsp30b* did not alter hyphal sensitivities to NaCl (Figure 5B). Moreover, based on their median lethal doses (LD_50_s; J cm^−2^), *Δhsp30a* and *Δhsp30b* exhibited 44.53% and 36.40% reduction in conidial tolerance to UV-B irradiation, respectively, compared with the WT estimate (Figure 5C).

Lastly, the single deletion of *hsp20*, *hsp30a*, and *hsp30b* alleviated fungal virulence. In standard bioassays, the median lethal action via the normal infection was delayed by 1.75 d in *Δhsp20*, 1.64 d in *Δhsp30a*, and 1.48 d in *Δhsp30b*, compared with the LD_50_ estimate averaged as 4.43 d for virulence to *Galleria mellonella* larvae caused by the WT (Figure 5D).

The increases in fungal sensitivities to oxidants, a fungicide, and UV-B exposure and the delay in the fungal virulence caused by the single deletion of *hsp20*, *hsp30a*, and *hsp30b* were largely or well restored by the targeted gene complementation, suggesting *hsp20*, *hsp30a*, and *hsp30b* played important but differential roles in sustaining antioxidant capacity, carbendazim tolerance, UV-B resistance, and virulence in *B. bassiana*.

## 3. Discussion

Generally, there is a comparable number of sHsps harboring conserved α-crystallin domain (ca. 3–5) in many filamentous fungi such as *Aspergillus*, *Penicillium*, *Fusarium*, and *Magnaporthe* [18]. *S. cerevisiae* harbors two sHsps containing conserved α-crystallin domains, namely Hsp26 and Hsp42, and the deletion of one sHsp did not influence the expression of the other sHsp [6]. In *B. bassiana*, three sHsps containing the α-crystallin domain are found and transcriptionally interrelated. The depressions of *hsp20* and *hsp30a* in *Δhsp30b* and the upregulation of *hsp30b* in *Δhsp20* indicated that *hsp30b* positively regulated the expression of *hsp20* and *hsp30a*, and *hsp20* negatively regulated the expression of *hsp30b* under normal and heat-shocked conditions (Figure 1A). Meanwhile, *hsp30* was a negative mediator of *hsp20* and a positive mediator of *hsp30b* under normal conditions because its deletion resulted in the upregulation of *hsp20* and the downregulation of *hsp30b* (Figure 1A). The altered expression of *hsp30a* in *Δhsp20* suggested that *hsp20* is a negative mediator of *hsp30a* under normal conditions but a positive mediator under heat-shocked conditions (Figure 1A). Additionally, our study unveiled that *hsp20*, *hsp30a*, and *hsp30b* played differential roles in sustaining asexual development and fungal adaptation to host and environmental stresses, as discussed below.

Among three deletion mutants grown on SDAY plates, only *Δhsp30b* displayed a moderate defect in hyphal growth and only *Δhsp30a* showed the reduction in conidial yields (Figure 2), implicating differential but dispensable roles for the studied sHsp genes in the fungal growth and conidiation of *B. bassiana*. Previously, disruption of *hsp24* in *Cryphonectria parasitica* resulted in a slow growth rate under standard growth conditions but showed no difference in the conidiogenesis [19]. A moderate growth defect on minimal media and smaller colonies on serum-containing agar were also observed in *C. albicans Δhsp21* [9]. However, the deletion mutants of *S. cerevisiae hsp26* or *hsp42*, and *U. maydis hsp20*, have no detectable effects on growth rates in rich or minimal media [7,10]. In *B. bassiana*, the defective growth of *Δhsp30b* could be simply attributable to the delayed germination of conidia, decreased efficiency of its utilizing of different carbon and nitrogen sources, and less tolerance to carbon or nitrogen starvation. The much sparser conidiophores in the 3-day cultures of *Δhsp30a* (Figure S2) might have resulted in reduced conidial yields.

Moreover, cell wall integrity is important for the defense against environmental insults in microbes. In this study, all *Δhsp20*, *Δhsp30a*, and *Δhsp30b* mutants demonstrated impaired cell wall integrity due to higher sensitivity to Congo red and altered cell wall fragility and components (Figure 3). Our results differed from the unchanged growth of *Δhsp21* under the stress of Congo red in *C. albicans* [9]. The impaired cell wall might increase cell sensitivity to the stresses of oxidation, fungicide, high temperature, and UV-B irradiation. Here, the absence of *hsp20*, *hsp30a*, or *hsp30b* resulted in retarded conidial germination and/or hyphal growth in response to high temperature, oxidants, and carbendazim (Figure 4). The total activities of SODs and CATs are crucial for *B. bassiana* in response to menadione and H_2_O_2_ [20,21,22], respectively, and their reduction coincided with the increase in sensitivities caused by the single deletion of three sHsp genes (Figure 4C,D). Additionally, the absence of *hsp30a* or *hsp30b* inhibited the germination of conidia after exposure to UV-B irradiation (Figure 5C). The decreased conidial thermotolerances and/or hyphal tolerances to high temperature caused by the deletion of *hsp20*, *hsp30a*, or *hsp30b* were similar to growth defects in *Δhsp24* of *C. parasitica* and *Δhsp21* of *C. albicans* [9,19] and the increases in conidial germination and hyphal growth in the transformants overexpressing *hsp25* in *Metarhizium robertsii* [23] but differed from no growth differences observed in *Δhsp26* and *Δhsp42* of *S. cerevisiae* and the transformants overexpressing *Trichoderma virens hsp23* in *T. harzianum* [6,7,24] under high temperature. The increased sensitivities to H_2_O_2_ and menadione were in agreement with the findings observed in *Δhsp20* of *U. maydis* and *Δhsp21* of *C. albicans* [9,10] but differed from the unchanged growth observed in *Δhsp24* of *C. parasitica* [19]. In addition, the deletion of each sHsp gene did not affect the hyphal sensitivities to NaCl in *B. bassiana* (Figure 5B), which was coincided with that reported in *Δhsp24* of *C. parasitica* [19] but different from the increased tolerance observed in *Δhsp21* of *C. albicans* [9].

Lastly, three sHsp genes contributed to fungal virulence in *B. bassiana* (Figure 5D). The attenuated pathogenicity through normal cuticle infection was consistent with that observed in *Δhsp24* of *C. parasitica* [19], *Δhsp21* of *C. albicans* [9], and *Δhsp20* of *U. maydis*. The efficient utilization of limited nutrients for the conidial germination and hyphal extension on the insect body surface is required for *B. bassiana* to penetrate through the host cuticle into the host haemocoel [25]. After entry into host haemocoel, *B. bassiana* conidia and hypha could suffer from superoxide anions generated from the defense immunity of the host after penetration [15]. Thus, the attenuated virulence might be owing to the reduction in antioxidant activities and/or changes in efficient utilization of limited nutrients caused by the deletion of *hsp20*, *hsp30a*, or *hsp30b* in *B. bassiana*.

## 4. Materials and Methods

### 4.1. Microbial Strains, Culture Conditions, and Chemicals

The WT strain of *B. bassiana* ARSEF 2860 (Bb2860) and its mutants were cultivated in SDAY consisting of 4% glucose, 1% peptone, 1% yeast extract, and 1.5% agar at 25 °C in a light/dark cycle of 12:12 h for normal growth and conidia germination. All the above strains were also grown in CZA containing 3% sucrose, 0.3% NaNO_3_, 0.1% K_2_HPO_4_, 0.05% KCl, 0.05% MgSO_4_, and 0.001% FeSO_4_ plus 1.5% agar or 1/4 SDAY (amended with 1/4 of each SDAY nutrient) for their responses to nutritional and chemical stresses at the same regime as SDAY. *Escherichia coli* DH5α (Tsingke Biological Technology Co., Ltd., Beijing, China) was cultured in Luria-Bertani (LB) medium at 37 °C for plasmid propagation. Additionally, all chemicals used in this study were analytical grade and purchased from Sigma (St. Louis, MO, USA) unless indicated otherwise.

### 4.2. Cloning and Analysis of Three sHSPs Containing Conserved α-Crystallin Domain in B. bassiana

Three α-crystallin domain-containing sHsps were found in the genome of *B. bassiana* Bb2860 [13] using Hsp26 and Hsp42 sequences of *S. cerevisiae*. The identified genes (tag code: BBA_07886 for *hsp20*, BBA_08688 for *hsp30a*, and BBA_02057 for *hsp30b*) were amplified from Bb2860 via PCR with designed primer pairs (Table S1) and sequenced at Invitrogen (Shanghai, China). The sequences of deduced proteins were structurally analyzed using online BLASTP procedure (http://blast.ncbi.nlm.nih.gov/blast.cgi (accessed on 20 March 2022)), followed by phylogenetic analysis with sHsp containing α-crystallin domain from other fungi in the NCBI protein database using a neighbor-joining method in MEGA 7.0 software (Mega Limited, Auckland, New Zealand). The molecular size and theoretical isoelectric point of the deduced proteins were predicted at https://web.expasy.org/protparam/ (accessed on 12 April 2022) and their subcellular localization was predicted at https://wolfpsort.hgc.jp/ (accessed on 13 April 2022).

### 4.3. Construction of hsp20, hsp30a, and hsp30b Mutants

*Hsp20*, *hsp30a*, and *hsp30b* were deleted by homologous replacement as described previously [17]. Briefly, 5′ and 3′ fragments of each gene were amplified from the extracted genomic DNA of WT and inserted into p0380-*bar* cut with appropriate restriction enzymes (NEB, Beijing, China), yielding three deleted plasmids of p0380-5′*hsp20*-*bar*-3′*hsp20*, p0380-5′*hsp30a*-*bar*-3′*hsp30a*, and p0380-5′*hsp30b*-*bar*-3′*hsp30b* for the deletion of *hsp20*, *hsp30a*, and *hsp30b*, respectively. The full-length coding sequences of *hsp20*, *hsp30a*, and *hsp30b* with its flanking regions were amplified from the genome of WT and ligated into the plasmid of p0380-*sur*-gateway to exchange for the gateway fragment under the action of Gateway^®®^ BP Clonase™ II Enzyme Mix (Invitrogen), forming complemented plasmids of p0380-*sur*-*hsp20*, p0380-*sur*-*hsp30a*, and p0380-*sur*-*hsp30b* for targeted gene complementation. All paired primers used for gene deletion and complementation are listed in Table S1. The deleted and complemented plasmids were transformed into the WT and corresponding deleted mutants, respectively, via *Agrobacterium*-mediated transformation [26]. Putative mutant colonies were screened by the *bar* resistance to ammonium glufosinate (200 μg/mL) and the *sur* resistance to chlorimuron ethyl (10 μg/mL), respectively, followed by the identification via PCR and qRT-PCR with paired primers (Table S1). Positive mutants and their control strains (WT and complemented mutants) were used in the experiments described below in three independent replicates.

### 4.4. Transcriptional Profiling of hsp20, hsp30a, and hsp30b

An aliquot of 100 μL of a conidial suspension of 10^7^ conidia mL^−1^ (the same used below unless specified) was initially spread on SDAY with cellophane and incubated at 25 °C and 12:12 h for 3 days. For a heat shock treatment, the 3-days-old hyphal cultures were exposed to 40 °C for 1 h. The total RNA was extracted from the cultures with RNAiso™ Plus Reagent (TaKaRa, Dalian, China) and reversely transcribed into cDNAs with PrimeScript^®®^ RT reagent kit (TaKaRa), respectively. The transcriptional levels of each gene were determined by qRT-PCR with paired primers (Table S1) under the action of SYBR^®®^ Premix Ex TaqTM (TaKaRa). The transcript of fungal γ-actin gene was used as an internal standard. The relative transcript levels of each gene in the deleted and complementary mutants were calculated based on the 2^−ΔΔCt^ method with respect to the WT standard [27].

### 4.5. Measurement of Growth and Conidiation

A suspension of 1 μL of 10^6^ conidia mL^−1^ was dropped on the plates (9 cm diameter) of SDAY, CZA, and modified CZA media with different carbon or nitrogen sources. The modified CZA media originated from the standard CZA by replacing sucrose with glucose, galactose, glycerol, fructose, trehalose, maltose, or acetate (NaAc) as the sole carbon source, replacing NaNO_3_ with NaNO_2_ or NH_4_Cl as the sole nitrogen source, and removing sucrose (carbon starvation) and NaNO_3_ (nitrogen starvation), respectively. After 8-day incubation at 25 °C, the diameter of each colony was recorded using two measurements taken perpendicular to each other across the center of colony. Additionally, SDAY plates with 1 μL of conidial suspension were also incubated at 34 °C and 12:12 h for 8 days, followed by measuring colony diameters as a growth index of each strain at the high temperature. All the experiments were conducted in triplicate.

For quantification of the capacity for conidiation, 100 μL of conidial suspension was spread evenly on SDAY plate (9 cm diameter) and cultured at 25 °C for 7 days. From day 4 onwards, three 5 mm-diameter plugs were randomly taken from each plate daily using a cork borer and immersed into 1 mL of 0.02% Tween 80. After the vibration and removal of hyphal debris by filtration, the number of conidia was measured using a hemocytometer and converted to the number of conidia per cm^2^ plate culture. All the experiments were conducted in triplicate.

To assess the quality and viability of conidia from SDAY plates, 50 μL of conidial suspension was uniformly spread on GM plates (6 cm diameter) consisting of 2% sucrose, 0.5% peptone, and 1.5% agar. Percentage germination was measured every 2 h under the microscopy. The time required for 50% conidial germination of each strain was calculated by model fitting analysis and was defined as GT_50_. All the experiments were conducted in triplicate.

### 4.6. Assays of Hyphal Responses to Chemical Stresses

100 μL of conidial suspension was spread on cellophane overlaid SDAY plates and incubated at 25 °C. After 3 days, hyphal discs (5 mm diameter) were cut off from the culture and attached to the plates (9 cm diameter) of 1/4 SDAY alone (control) or 1/4 SDAY supplemented with a concentration of Congo red (0.5–3 mg/mL), menadione (2–8 mM), H_2_O_2_ (20–80 mM), carbendazim (0.4–2 μg/mL), and NaCl (0.4–2 M). All plates were cultured at 25 °C for 6 d. The diameter of each colony was measured and an EC_50_ for each chemical to inhibit 50% of the colony growth was calculated by modeling analyses of relative growth indices of each strain over the chemical gradient. All the experiments were conducted in triplicate.

### 4.7. Assays for Conidial Virulence and Tolerance to Various Stresses

100 μL of conidial suspension of each strain was evenly spread on GM plates alone (control) and containing 1.0 mg/mL Congo red, 0.2 mM menadione, or 4 mM H_2_O_2_, 1.2 M NaCl. After 24 h incubation at 25 °C, the relative germination was calculated as the ratio of percent germination under each stress over that in the control. Conidial thermotolerance and UV-B resistance were quantified as LT_50_ after exposure to 45 °C wet heat for 0–120 min and LD_50_ after exposure to UV-B irradiation at 0–0.8 J cm^−2^, as described previously [21]. Conidial virulence was assayed on *G. mellonella* larvae by normal cuticle infection, as described previously [17]. All the experiments were conducted in triplicate.

### 4.8. Assessment of Cell Wall Integrity and Antioxidant Enzymes Activities

Several methods were used to reveal the differences in cell wall integrity in the WT and mutants. First, the fragility of the cell wall was assessed using a method of cell wall degradation [28]. Hyphal cells (~100 mg) from the 2-day-old agar-free SDAY cultures were washed twice with PBS (pH 7.0) and resuspended in 2 mL of 0.8 M sucrose containing snailase and lysing enzyme (Sigma) of 10 mg/mL. After 6 h incubation at 37 °C, cell wall lysing was terminated by keeping the cell suspension in ice. The concentration of protoplasts released from the hyphal cells of each suspension was quantified as an index of the cell wall fragility using a haemocycometer. Second, carbohydrate epitopes on the surfaces of conidia were probed by the method of lectin binding assay [29]. Briefly, conidia were fixed in 3% formaldehyde overnight at 4 °C, washed three times with PBS buffer, and then resuspended in the buffer. The pretreated conidia were suspended in the appropriate lectin-binding buffer for 1 h labeling with the Alexa fluor 488-labeled GNL specific to mannose residues and WGA specific to β-GlcNAc and sialic acids from Molecular Probes-Invitrogen and Vector laboratories in darkness following the user’s guide. After the removal of unbound lectin, fluorescent signals in every 2 × 10^4^ labeled conidia were quantified on the flow cytometer FC 500 MCL (Beckman Coulter, Brea, CA, USA) using an argon laser at the excitation/emission wavelengths of 488/530 nm. Finally, cell walls of aerial conidia were examined via transmission electron microscopy as described previously [30].

For antioxidant enzyme activities, the total activity (U mg^−1^) of SODs and CATs were assayed as described previously [15]. All the experiments were conducted in triplicate.

### 4.9. Statistical Analyses

Results from three replicates were expressed as mean ± standard deviation (SD) and subjected to a one-factor (strain) analysis of variance (ANOVA) and Tukey’s honestly significant difference (HSD) test. Results were statistically significant when *p* < 0.05 in all experiments.

## 5. Conclusions

In summary, three sHsps containing conserved α-crystallin domain exist in the genome of *B. bassiana* and are transcriptionally interrelated under normal and heat-shocked conditions. Moreover, *hsp20*, *hsp30a*, and *hsp30b* played important but differential roles in sustaining cell wall integrity, antioxidant activity, fungal tolerances to high temperature and carbendazim fungicide, and fungal virulence in *B. bassiana*. Additionally, *hsp30a* positively regulated fungal conidiation and conidial tolerance to UV-B irradiation, and *hsp30b* positively contributed to hyphal growth in rich and minimal media and conidial germination in response to UV-B irradiation. These findings unveil possible means of improving field persistence and efficacy of a fungal formulation by manipulating the sHsp genes in a candidate strain.

## Data Availability

The datasets generated during and/or analyzed during the current study are available from the corresponding author on reasonable request.

## Supplementary Materials

The following supporting information can be downloaded at: https://www.mdpi.com/article/10.3390/ijms23126717/s1.

## Abbreviations

sHsps	Small Heat Shock Proteins
WT	wild type
CZA	Czapek-Dox agar
SDAY	Sabouraud dextrose agar
GM	germination medium
LB	Luria-Bertani
qRT-PCR	quantitative real-time PCR
NaAc	acetate
LT_50_	median lethal time
LD_50_	median lethal dose
GNL	Galanthus nivalis lectin
WGA	wheat germ agglutinin
SODs	superoxide dismutases
CATs	catalases
SD	standard deviation
ANOVA	analysis of variance
HSD	Tukey’s honestly significant difference
CBD	carbendazim
