# Supplementary material for: Differential Roles of Three α-Crystallin Domain-Containing sHsps of Beauveria bassiana in Asexual Development, Multiple Stress Tolerance and Virulence
Source: Int J Mol Sci. 2022 Jun 16;23(12):6717. doi: 10.3390/ijms23126717 (PMC9224193; doi:10.3390/ijms23126717)
Supplement: Supplementary file 1 [file ijms-23-06717-s001.zip › ijms-1724210-supplementary.pdf]

Journal: International Journal of Molecular Sciences

Differential Roles of Three α-Crystallin Domain-Containing sHsps of *Beauveria bassiana* in Asexual Development, Multiple Stress Tolerance and Virulence

Gang Zhou [1,2], Sheng-Hua Ying[3], Ming-Guang Feng [3,*], Jie Wang [1,*]

[1] College of Food Science, South China Agricultural University, Guangzhou, 510642, People's Republic of China

[2] Guangdong Provincial Key Laboratory of Microbial Culture Collection and Application, State Key Laboratory of Applied Microbiology Southern China, Institute of Microbiology, Guangdong Academy of Sciences, Guangzhou, 510070, People's Republic of China

[3] Institute of Microbiology, College of Life Sciences, Zhejiang University, Hangzhou, 310058, People's Republic of China

Gang Zhou: zgbees@gdim.cn

Sheng-Hua Ying: yingsh@zju.edu.cn

Ming-Guang Feng: mgfeng@zju.edu.cn

Jie Wang: jiewang@scau.edu.cn

* **Corresponding author.** Ming-Guang Feng: mgfeng@zju.edu.cn, College of Life Sciences, Zhejiang University, Hangzhou, 310058, People's Republic of China; Jie Wang: jiewang@scau.edu.cn, College of Food Science, South China Agricultural University, Guangzhou, Guangdong, 510642, People's Republic of China

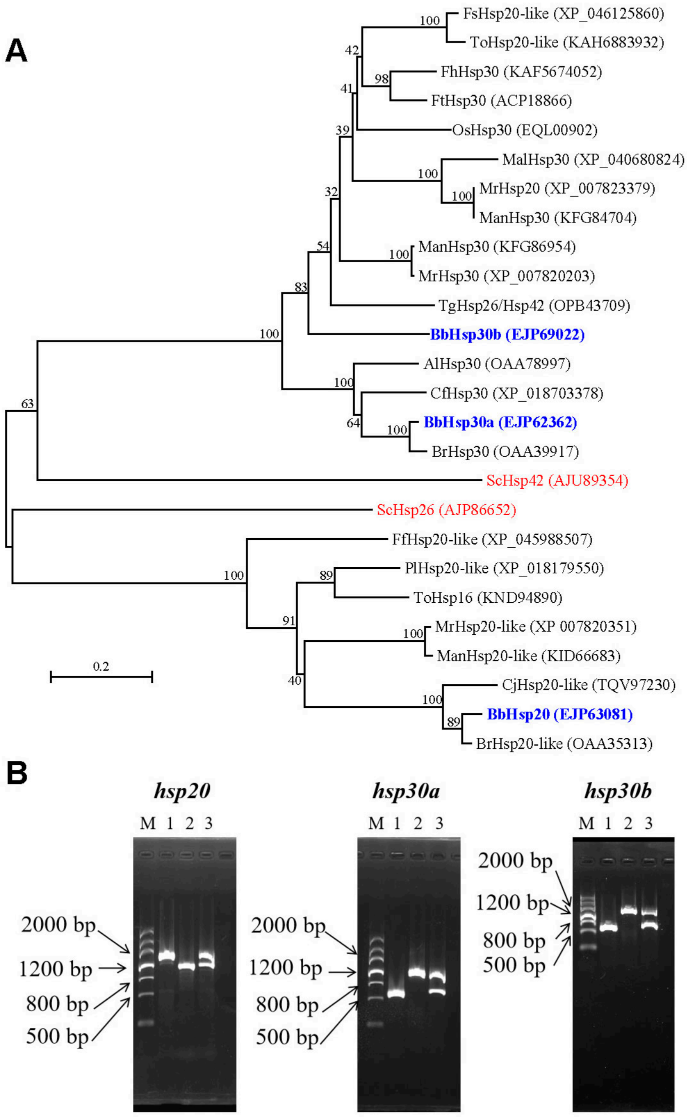

Figure S1. Phylogenetic analysis of three α-crystallin domain-containing sHsp genes found in the genome database of *B. bassiana* and identification of their mutants. (**A**) Phylogenetic tree for three sHsps of *B. bassiana* and other fungi based on a neighbor-joining method in MEGA7. The NCBI accession code of a given protein are given in the parentheses following each fungal name. Scale bar: branch length proportional to genetic distance. (**B**) The *hsp20*, *hsp30a* and *hsp30b* mutants identified via PCR. *Lane 1*: wild-type strain. *Lane 2*: deletion mutant. *Lane 3*: complemented mutant.

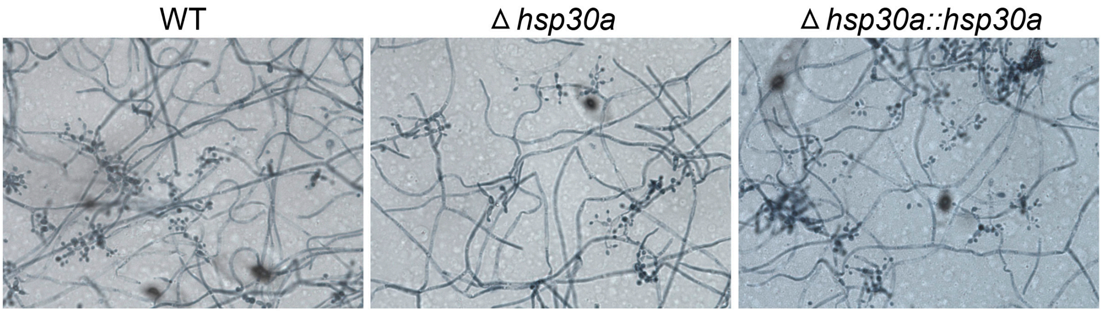

Figure S2. Conidiophores and conidia after conidia were cultivated on SDAY plates for 3 days, and visualized at 400 × magnification.

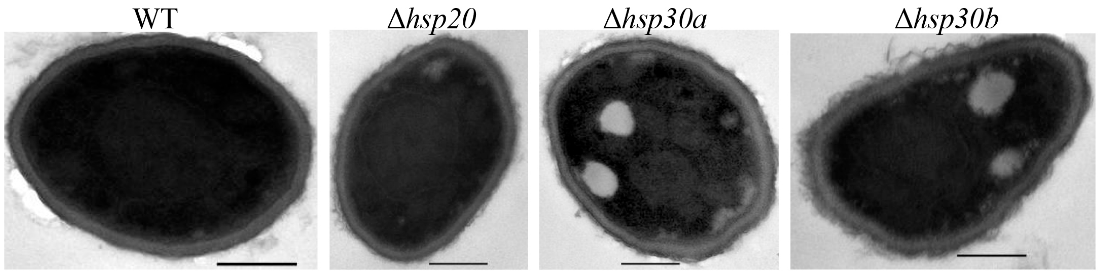

Figure S3. Images for the ultrathin sections of conidia observed under transmission electron microscopy (TEM).

Table S1 The paired primers used for the manipulation of *hsp20*, *hsp30a*, and *hsp30b* in this study

Name	Paired sequences (5’-3’)*	Purpose
hsp20-F/R	ATGGCCCCCACGCAACAACC / TCACAGAATGTCCACCTTGCG	Amplification of *hsp20*
hsp30a-F/R	ATGTCTCTTTTCCCCCG / GTTGATAGCAATGCGTCG	Amplification of *hsp30a*
hsp30b-F/R	ATGGCTTCCTTCTTTCCCC / GTTGATGCTGATGCGGAC	Amplification of *hsp30b*
hsp20up-F/R	AAAAAGGATCCTCATTGCCTCCATCCATACCG / AAAAAAAGCTTCGAGTTTGGTTGTTGCGTGG	Cloning *hsp20* 5 -end (2135 bp)
hsp20dn-F/R	AAAAAAGATCTATGATGTGATGATGCGTGCTG / AAAAAGTTAACTCTCGCCCCATCTGTCTACG	Cloning *hsp20* 3 -end (1537 bp)
hsp30aup-F/R	AAAAAGGATCCTCGCGCTAATTTTTGGTACTAAGGT / AAAAAGAGCTCTGTTTGCAGTGGTTGTAGTCTTGAT	Cloning *hsp30a* 5 -end (1406 bp)
hsp30adn-F/R	AAAAAAGATCTTGTTTCGGACAAGCCAACCATC / AAAAAACTAGTTCATATCTTCTTTGGGCCATCTCAC	Cloning *hsp30a* 3 -end (1374 bp)
hsp30bup-F/R	AAAAAGGATCCGTCATACTCCGATTGATTTTGG / AAAAACTGCAGAGTGGTGTTGTGTTTGATGTTG	Cloning *hsp30b* 5 -end (1411 bp)
hsp30bdn-F/R	AAAAAAGATCTAGCAGCAGAACCAGCAGC / AAAAAACTAGTTGGTAAATCCCATAGGAACAAG	Cloning *hsp30b* 3 -end (1543 bp)
hsp20fl-F/R	*GGGGACCACTTTGTACAAGAAAGCTGGGT*TGTATTCCGTCTTTGTCTCTG / *GGGGACAAGTTTGTACAAAAAAGCAGGCT*TAGAAGAAGTGTCCGACGAGT	Cloning full-length *hsp20* (3915 bp)
hsp30afl-F/R	*GGGGACCACTTTGTACAAGAAAGCTGGGT*GAATGCCTACACTGTGGACT / *GGGGACAAGTTTGTACAAAAAAGCAGGCT*TGATTCGTAAGGAAGCCAGT	Cloning full-length *hsp30a* (2763 bp)
hsp30bfl-F/R	*GGGGACCACTTTGTACAAGAAAGCTGGGT*GATGACTCGGCACGAGGAAC / *GGGGACAAGTTTGTACAAAAAAGCAGGCT*TTTGTCAGTCCAGCGATGCC	Cloning full-length *hsp30b* (3478 bp)
pHsp20-F/R	CACAGGAAGTTGATACCGAAAT / GTCAAGATGGAGATAGTTGTGGTT	PCR detecting *hsp20*
pHsp30a-F/R	CAAACATCAAGACTACAACCACTG / CTTAGTTGATAGCAATGCGTCG	PCR detecting *hsp30a*
pHsp30b-F/R	ACAACATCAAACACAACACCAC / TGTTTAGTTGATGCTGATGCGG	PCR detecting *hsp30b*
qHsp20-F/R	TCAGGAGATGAGAAGAAC / GCTGTGATGATGTTTGTT	qRT-PCR detecting *hsp20*
qHsp30a-F/R	TCTTCCCTTGGTCGTGAT/GTATTGGTCGCTGCTCTC	qRT-PCR detecting *hsp30a*
qHsp30b-F/R	ATGGCTTCCTTCTTTCCC/AGTATGTGTCAAACTCGTC	qRT-PCR detecting *hsp30b*

* The underlined region indicates the restriction site (*hsp20*: *Bam*HI/*Hin*dIII and *Bgl*II/*Hpa*I; *hsp30a*: *Bam*HI/*Sac*I and *Bgl*II/*Spe*I; *hsp30b*: *Bam*HI/*Pst*I and *Bgl*II/*Spe*I; The italic underlined area indicates the recognition sequence of the gateway exchange reaction.
